# Cataloging Actionable Pharmacogenomic Variants for Indian Clinical Practice: A Scoping Review

**DOI:** 10.3390/jox15040101

**Published:** 2025-07-01

**Authors:** Sacheta Sudhendra Kulkarni, Venkatesh R, Anuradha Das, Gayatri Rangarajan Iyer

**Affiliations:** 1 Tata Institute for Genetics and Society, Gandhi Krishi Vignana Kendra Campus, Bellary Road, Bengaluru 560065, Karnataka, India; sacheta.kulkarni@tigs.res.in (S.S.K.); venkatesh.r@tigs.res.in (V.R.); ritzanuradha@gmail.com (A.D.); 2 Dr. D. Y. Patil Biotechnology and Bioinformatics Institute, Tathawade, Pune 411033, Maharashtra, India

**Keywords:** pharmacogenomics, PharmGKB, IndiGenomes, adverse drug reactions, pharmacogenomics testing, gene–drug interaction, indigenous data

## Abstract

Background: Pharmacogenomics (PGx), a pivotal branch of personalized medicine, studies how genetic variations influence drug responses. Despite its transformative potential, the adoption of PGx in Indian clinical practice faces challenges, such as the lack of population-specific data, evidence-based guidelines, and complexities in interpreting genomic reports. Comprehensive datasets tailored to Indian patients are essential to facilitate the integration of PGx into clinical settings. Methodology: The study collates pharmacogenomic data from multiple sources, including essential drugs listed by the World Health Organization (WHO), drugs used in neonatal intensive care units (NICUs), minimum sets of alleles recommended by the Association for Molecular Pathology (AMP), and catalogs the allele frequencies from the IndiGenomes database to address gaps in actionable PGx for the Indian population. Curated datasets were used to identify pharmacogenomic variants relevant to clinical practice. Results: Overall, 24 prime genes are essential for the outcomes of 57 drugs. In adults, 18 genes influence the metabolism of 44 drugs whereas, in pediatric populations, genotypes of 18 genes significantly impact the metabolism of 18 drugs. Two over-the-counter drugs with actionable PGx variants were identified: ibuprofen and omeprazole. These findings emphasize the clinical relevance of PGx for commonly used drugs, underscoring the need for population-specific data. Conclusions: As the data of several Indian human genome projects become available, an overarching need exists to establish and regulate the dynamic actionable PGx in Indian clinical practice. This will facilitate the integration of pharmacogenomic data into healthcare, enabling effective and personalized drug therapies.

## 1. Background

Pharmacogenomics (PGx) studies how genes affect the response of an individual to drugs. This field combines pharmacology (the science of drugs) and genomics (the study of genes and their functions) to develop effective, safe medications that can be prescribed based on the genetic makeup of an individual. Unlike traditional medicine, which adopts a one-size-fits-all approach to drug therapy, PGx aims to personalize treatment regimens on the basis of the genetic profile of an individual.

PGx studies the genomic variations responsible for differences in drug metabolism like pharmacokinetics (what happens to the drug once inside the body), and pharmacodynamics (how the drug affects the body), leading to varied drug responses among individuals [1,2,3]. Every physiological and pathological process requires considerable enzymatic machinery to carry out the designated functions [4]. Drug metabolism is carried out by key proteins associated with absorption, distribution, bioactivation, metabolism and excretion of metabolites [5]. Variation in these enzymes determines their duration of action and toxicity [3,6]. Based on the phenotypic activity, individuals can be classified as poor, intermediate, normal, rapid, and ultra-rapid metabolizers [7,8]. In the last several years, with concerted knowledge from patient outcomes, functional assays, and pharmacokinetic and pharmacodynamic studies, researchers have been able to associate characteristic genotypes/diplotypes with their phenotypes [9]. To illustrate, *CYP2C19* is a gene associated with the metabolism of proton pump inhibitors (PPI) [10]. At the genotype level, CYP2C19 *1 is known to be associated with normal metabolism, *3 is associated with reduced enzyme activity, and *17 with rapid enzyme activity. Hence, an individual with the CYP2C19 *1/*1 diplotype will be a normal metabolizer, one with CYP2C19 *1/*3 is an intermediate metabolizer, one with CYP2C19 *3/*3 is a poor metabolizer, one with CYP2C19 *1/*17 is a rapid metabolizer, and one with CYP2C19 *17/*17 is ultra-rapid metabolizer [8]. These individuals must be prescribed PPI based on their diplotype for optimal response [10]. These insights can assist medical professionals in anticipating the drug response of an individual to a specific medication, enabling customized and safer treatment plans [1,6,11].

From the formal origin of pharmacogenomics in the 1950s, to the human genomics project in the early 2000s until today, several studies have listed the evidence of genes involved in drug response impacting clinical outcomes [7,8,12,13]. There are established consortia, regulatory bodies and agencies like the Pharmacogenomics Research Network (PGRN), the Clinical Pharmacogenetics Implementation Consortium (CPIC), the U.S. Food and Drug Administration (FDA), the Ubiquitous Pharmacogenomics Consortium (UPGx), and the International Society of Pharmacogenomics (ISPG) that oversee and promote the clinical actionability of pharmacogenomic profiling for safe and effective medication [13].

The clinical utility of PGx has been well-documented in various regions; however, data specific to the Indian population are sparse. India’s genetic diversity, coupled with distinct population substructures, presents a unique challenge in determining how relevant pharmacogenomic variants impact therapeutic outcomes. Moreover, global pharmacogenomic databases are often built on data from populations in Europe and North America, leading to gaps in the applicability of these findings to Indian patients [14]. This underscores the need for population-specific studies that can identify actionable pharmacogenomic variants relevant to Indian healthcare.

Previous studies have highlighted the importance of incorporating pharmacogenomic data into treatment protocols, with examples of genetic polymorphisms significantly affecting drug metabolism and efficacy [10,11,12,13]. There is literature on how pharmacogenomics can be incorporated into hospital records as either reactive or preemptive testing [15], on a population scale [16], and bydisease–drug specific guidelines [10] in different parts of the globe. However, the lack of comprehensive datasets tailored to Indian patients hinders the full integration of PGx into Indian clinical practice. This study seeks to address that gap by cataloging pharmacogenomic variants from key sources, including essential drugs listed by the World Heath Organization (WHO), drugs critical for neonatal care in the neonatal intensive care unit (NICU), and the minimum set of alleles recommended by the Association of Molecular Pathology (AMP).

By creating a comprehensive repository of pharmacogenomic variants relevant to the Indian population, this study aims to bridge the gap between pharmacogenomic research and its practical application in clinical settings. This approach could significantly enhance the safety and efficacy of drug prescription in India, advancing personalized medicine and improving patient outcomes.

## 2. Objectives

### 2.1. To Catalog the PGx of WHO Essential Medicines

1. What is the total number of drugs and the diseases they address, considered essential by WHO in adult and pediatric groups?

2. How many drugs from 2.1.1 have recognized and actionable pharmacogenomics evidence?

3. What is the frequency of the actionable pharmacogenomic variants in the Indian population?

4. How many of the drugs are available over the counter in India?

### 2.2. To Catalog the PGx of NICU Medicines

1. What is the total number of drugs used in NICUs, including Indian literature?

2. How many drugs from 2.1.1 have pharmacogenomic evidence under the pediatric focus of PharmGKB?

3. What is the frequency of relevant variants in the Indian population?

4. Are there established adverse drug reactions incidents associated with these drugs?

5. Can these adverse drug reactions be correlated with infant mortality rate?

### 2.3. To Enlist the Critical Genes That Require Initial Testing for Drug Adverse Effects for Personalized Medicine

1. What are the alleles/variants testing is recommended by the Association of Molecular Pathology for pharmacogenomics?

2. What is the frequency of c.1 alleles in the Indian population?

3. Which are the drugs impacted by 2.3.1 and 2.3.2?

4. Considering the findings (2.1.3, 2.1.4, 2.2.3, 2.2.4, 2.3.2, and 2.3.3), what should be common alleles recommended for the Indian population for preemptive and reactive pharmacogenomic studies?

## 3. Methods

**Figure 1:**
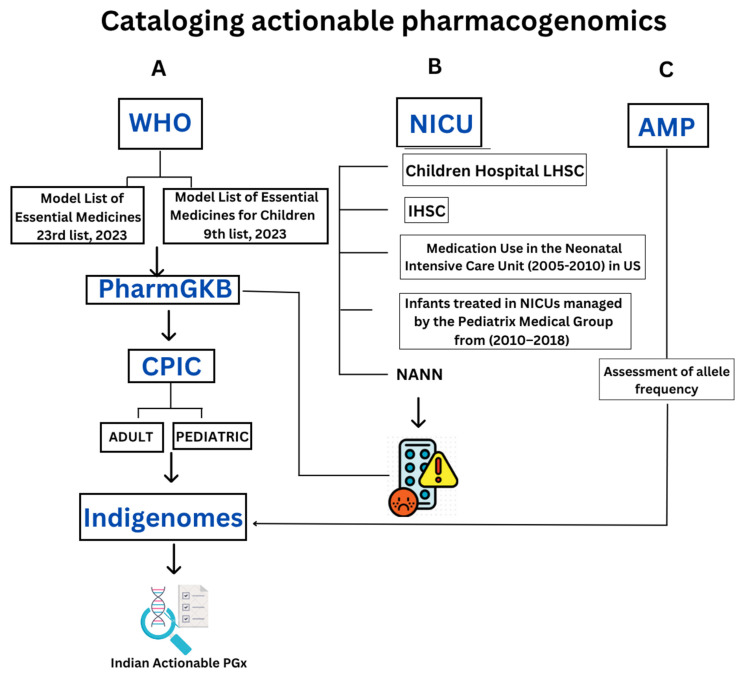
Study workflow. Three objectives were compiled for cataloging actionable pharmacogenomics. (**A**) World Health Organiszation list of essential drugs for adult and pediatrics were compiled, and their drug–gene information from Pharmacogenomics Knowledgebase (PharmGKB) were collated. Based on Clinical pharmacogenetics implementation consortium (CPIC) level 1 and 2, further drugs were shortlisted and frequencies from the IndiGenomes database were collated. (**B**) Neonatal Intensive Care Unit (NICU) medication list was compiled from five sources; their adverse drug reaction was recorded followed by PharmGKB analysis. (**C**) The Association for Molecular Pathology (AMP) recommended minimum set of alleles were recorded along with their allele frequency and IndiGenomes data.

The study aims to achieve three primary objectives: cataloging the actionable PGx of the WHO’s essential drugs, cataloging PGx of drugs used in NICUs, and collating the allele frequencies of the minimum set of alleles for PGx testing based on AMP guidelines in the Asian and Indian context. Refer to Figure 1 for a brief overview of relevant data from the Pharmacogenomics Knowledgebase (PharmGKB), CPIC, and IndiGenomes websites, systematically collected and compiled using a Microsoft excel for further analysis.

### 3.1. Essential Drugs from WHO

#### 3.1.1. Obtaining Drug Data from the WHO Website and WHO List of Essential Drugs for Children

We accessed the WHO website and retrieved the WHO Model List of Essential Medicines—23rd list, 2023. As per WHO, “Essential medicines are those that satisfy the priority health care needs of a population. They are selected with due regard to disease prevalence and public health relevance, evidence of efficacy and safety, and comparative cost-effectiveness. They are intended to always be available in functioning health systems, in appropriate dosage forms, of assured quality and at prices individuals and health systems can afford” [17]. For pediatrics, we followed the WHO Model List of Essential Medicines for Children—9th list, 2023 [18].

#### 3.1.2. Identifying Gene Pairs, Star Alleles, and Reference Single Nucleotide Polymorphism ID Number (rsIDs)

PharmGKB offers curated data, tools, and resources to facilitate the translation of PGx results into clinical practice by leveraging guidelines from the CPIC, the Dutch Pharmacogenetics Working Group (DPWG), and the recommendations from the FDA, along with other PGx guidelines [19]. We navigated through the PharmGKB website to identify relevant drug–gene associations. This was repeated for drugs in pediatrics with the filter of “pediatric focus”.

DPWG [20] develops pharmacogenetic guidelines to support healthcare professionals in prescribing medications based on the genetic profile of an individual. The CPIC [21] level refers to the categorization of pharmacogenetic information into four levels based on the strength of evidence and clinical actionability. Level A represents strong evidence supporting the gene–drug interaction, indicating a significant impact on drug dosing or selection. Level B has moderate evidence, with some variability in clinical utility or the strength of evidence. Level C shows weak evidence, necessitating cautious interpretation and limited clinical utility. Finally, Level D denotes insufficient evidence, and such gene–drug interactions are typically not recommended for routine clinical use.

These guidelines guide dose adjustments or alternative therapies to improve drug efficacy and safety. The FDA biomarkers are part of the U.S. Food and Drug Administration’s efforts to incorporate biomarker information into drug labeling [22]. This helps in identifying genetic markers that can predict drug response, adverse reactions, or disease susceptibility, thereby aiding personalized medicine.

The drug-associated star alleles and rsIDs for each gene variant were documented.

#### 3.1.3. Checking PharmGKB Score and CPIC Level

The clinical annotations of several points of evidence and guidelines are assigned scores by PharmGKB. Levels 1 and 2 have high and moderate evidence and thus can be utilized for taking informed decisions by clinicians to avoid adverse drug reactions. The CPIC level assigned to each drug–gene interaction indicates the strength of evidence and recommendations provided by CPIC guidelines regarding the clinical utility of PGx information. All level 1 and 2 (1A, 1B, 2A and 2B) gene–drug associations are considered to be medically actionable, thus their global frequencies were retained for further analysis.

#### 3.1.4. Extracting Minor Allele Frequency (MAF) Data from IndiGenomes

The IndiGenomes [23] website was used to access data from the Indian 1000 Genomes Project. IndiGenomes hosts allele frequencies and genetic variations among India’s indigenous populations, providing a unique resource for studying population-specific genetic variations. This focuses on the Indian population to obtain specific minor allele frequencies information relevant to the study.

#### 3.1.5. Data Analysis and Interpretation

The compiled data was analyzed using the Microsoft Excel application to identify trends, patterns, and associations between drug–gene interactions and population-specific genetic variations. The findings in the context of personalized medicine and the potential implications for clinical practice, established drug safety, and efficacy were interpreted. For visualization, an in-house Python (Version 3.12.5) script was developed.

### 3.2. PGx of NICU Drugs

#### 3.2.1. Obtaining Drug Data from Different NICU Medication Databases

Data about drug prescriptions were collected from the NICU section of the London Health Sciences Centre (LHSC) [24], IOWA Health Care [25], publications such as “Medication Use in the Neonatal Intensive Care Unit (2005–2010) in the United States” [26], infants treated in NICUs managed by the Pediatrix Medical Group from (2010–2018) [27], and high-risk neonatal medication examples provided in “Medication Safety in the NICU” by the National Association of Neonatal Nurses (NANN) [28].

#### 3.2.2. Identification of Drug Names and Prescriptions

Drug names and prescriptions mentioned on the NICU website, and articles were identified and documented. This included both generic and brand names of drugs administered to neonatal patients within the NICU databases and the publications.

#### 3.2.3. Assessment of Adverse Drug Reaction (ADR)

A comprehensive search was conducted to identify studies, clinical trials, and pharmacovigilance databases reporting ADRs associated with the drugs mentioned on the LHSC NICU, IOWA Health Care list and Medication Use in the Neonatal Intensive Care Unit (2005–2018), and high-risk neonatal medication examples provided in “Medication Safety in the NICU” by the NANN.

Data on the type, frequency, severity, and management of ADRs specific to drugs used in the NICU were extracted and recorded.

The subsequent steps are the same as steps 3.1.2 to 3.1.5 of the previous objectives.

### 3.3. Minimum Set of Alleles for PGx Testing Based on AMP Guidelines

The AMP [29] guidelines provide recommendations and standards for molecular diagnostic testing, including pharmacogenetic testing. These guidelines offer guidance on various aspects of testing, such as assay validation, result interpretation, and reporting. They aim to ensure high-quality and accurate molecular testing practices, ultimately contributing to improved patient care and clinical outcomes. The AMP minimum set of alleles is practiced in clinical environments where genetic testing is used to inform drug prescribing and management, with guidelines and support from resources like PharmGKB.

The Minimum Set of Pharmacogenetic Alleles is a curated list of genetic variants that have significant clinical relevance in PGx. These alleles are crucial for guiding drug therapy decisions based on the genetic makeup of an individual, enabling healthcare providers to tailor drug choices and dosages to optimize efficacy and minimize adverse effects. We also analyzed the list to assess the feasibility of preemptive testing for the minimum set of genes to be tested for over-the-counter and common medicines.

#### 3.3.1. Compile the Gene List Recommended by AMP and Enlist the Drugs Affected by Allele Polymorphisms to Have a Varied Response

The list of gene–allele information prescribed to be tested by AMP was recorded. A list of drugs related to the identified gene variants was compiled. Drugs werecategorized based on allele function, ensuring a comprehensive understanding and application in clinical practice.

#### 3.3.2. Assessment of Allele Frequency

Allele frequency tables from databases such as PharmGKB were recorded to determine the frequency of alleles in different populations.

The subsequent steps are the same as steps 3.1.4 to 3.1.5 of the objectives.

## 4. Results

### 4.1. Essential Drugs from WHO

Overall, 726 drugs for adults and 370 drugs for children are listed by the WHO as essential. Their PharmGKB data and the results of adult and pediatric actionable drug–gene pair along with global frequencies are compiled in Supplementary Table S1.

#### 4.1.1. WHO Model List of Essential Medicines—23rd List, 2023

Data was curated from approximately 726 drugs listed in the WHO database, identifying a subset of 366 drugs with PharmGKB data. These drugs were then categorized based on their CPIC level of evidence.

**Figure 2:**
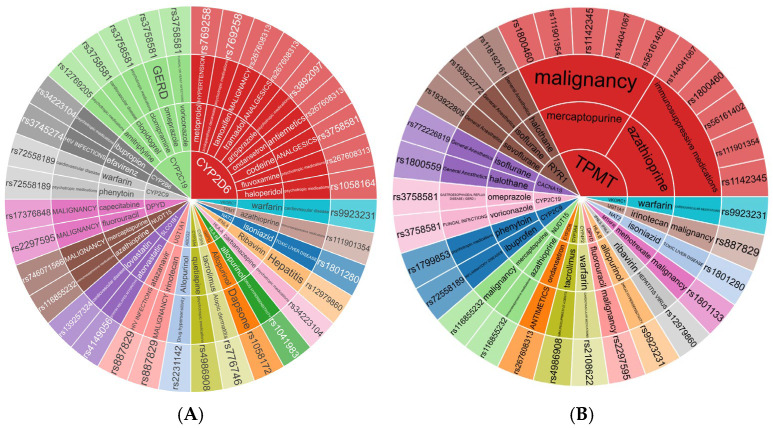
(**A**) Drugs with CPIC Level 1 and 2 evidence for adults with associated genes and medical conditions. (**B**): Drugs with CPIC Level 1 and 2 evidence for children with associated genes and medical conditions. The interactive HTML files are in Supplementary Graphs S1 and S2.

The study categorizes drugs into three evidence levels using CPIC guidelines: Level 1 (53 drugs) with solid evidence, Level 2 (12 drugs) with moderate support as given in Figure 2A,B (Interactive graph given as Supplementary Graph S1 and Graph S2), and Level 3 (161 drugs) with insufficient or lower-quality studies.

Level 1 and 2 drugs were further categorized based on the diseases they address, providing a detailed understanding of their therapeutic indications and medical relevance. The disease-focused classification of Level 1 and Level 2 evidence drugs with Indian data (Table 1) revealed their targeted therapeutic applications across a range of medical conditions. About 44 drugs with Level 1 and Level 2 evidence, supported by Indian data, are curated in Table 1.

#### 4.1.2. WHO Model List of Essential Medicines for Children—9th List (2023)

The WHO Model List of Essential Medicines for Children comprises a similar set of drugs to those found in the WHO List of Essential Medicines. This list includes 370 total drugs of which 95 of them had PharmGKB information on pharmacogenomic variants.

These drugs were then categorized based on their level of evidence: Level of Evidence 1 consists of 16 drugs; Level of Evidence 2 comprises 10 drugs as mentioned in Figure 2B (Interactive graph given as Supplementary Graph S2), and Level of Evidence 3 consists of 69 drugs.

The disease-focused classification of Level 1 and Level 2 evidence drugs with Indian data (Table 1) revealed their targeted therapeutic applications across a range of medical conditions.

### 4.2. PGx of NICU Drugs

The study compiled data on drugs commonly used in NICUs and assessed their adverse effects, associated genes, rsIDs, level of evidence sourced from the PharmGKB database, and Indian frequency from the IndiGenomes website, totaling approximately 181 unique drugs from five sources as previously mentioned.

**Figure 3:**
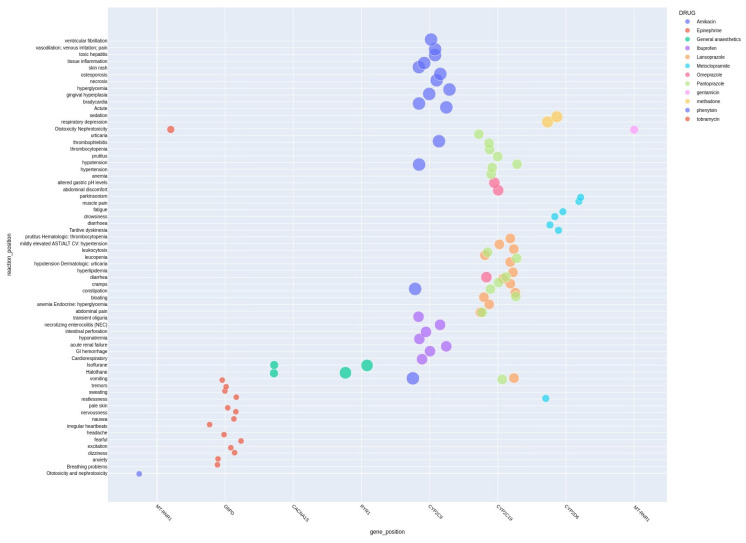
Bubble plot of adverse drug effects of Level 1 and Level 2 NICU drugs along with the genes associated with them.

Figure 3 displays the bubble plot of adverse drug effects of Level 1 and Level 2 NICU drugs along with the genes associated with them. Table 2 provides the drug list, and associated adverse drug reactions along with Level 1 and Level 2 pharmacogenomic evidence in NICU drugs. The identified adverse effects of NICU drugs are illustrated in Supplementary interactive Graph S3 and Supplementary Table S2. 

### 4.3. Minimum Set of Alleles for PGx Testing Based on AMP Guidelines

The AMP PGx Working Group’s guidelines categorize PGx alleles into Tier 1 (mandatory) and Tier 2 (optional) based on rigorous criteria. The AMP PGx Working Group’s guidelines have curated a total of nine genes into Tier 1 (mandatory) and Tier 2 (optional) categories for PGx testing. Their global and Indian frequencies along with the number of drugs they impact are listed in Table 3. The 24 prime genes that we propose for pre-emptive testing to assist clinicians in delivering personalized medicine are listed in Table 4.

## 5. Discussion

Pharmacogenomics is a translational specialty that strives to improve drug response, maximizing benefits while minimizing side effects for optimal healthcare outcomes. By examining essential drugs listed by the WHO, exploring PGx of NICU drugs, and evaluating the minimum set of alleles for PGx testing based on AMP guidelines, this scoping review contributes to a dynamic compilation of actionable PGx for common drugs to improve healthcare outcomes.

From the WHO Essential Medicine (N = 726) Level of Evidence 1 status was given to 53 drugs, and Level of Evidence 2 was given to 12 drugs. About 359 drugs did not have PharmGKB information and 161 drugs had Level 3. This disparity underscores the need for more research globally to make informed clinical decisions. Approximately 24 drugs with Level 1 and Level 2 evidence had missing MAF data in the IndiGenomes, demonstrating the direction of further active pharmacogenomics studies to be conducted in India.

From the essential medicines list for children (N = 370), about 26 drugs, Level 1 (16 drugs), and Level 2 (10 drugs) denoted higher confidence in efficacy and safety and thus can be applied for guiding pediatric care. Approximately nine drugs with Level 1 and Level 2 evidence had missing MAF data from IndiGenomes. Continuous updates and research are necessary to refine the evidence base, especially for drugs with no evidence or Level 3 evidence, ensuring comprehensive pediatric treatment options.

About 57 drugs with actionable pharmacogenomics relevant to India address various conditions, including mental health (amitriptyline, carbamazepine, phenytoin), autoimmune diseases (azathioprine), and cancer (fluorouracil, mercaptopurine, nilotinib, methotrexate, rituximab, irinotecan). The dataset also covers medications for anesthetics, liver diseases, and autoimmune disorders, aiding clinical decision-making and public health policies. This indicates the need for medical education, formulation of guidelines, and policies for PGx-supported prescribing rationale for better healthcare outcomes.

The neonatal period, encompassing the initial 28 days post-birth, represents the most critical phase for infant survival, during which the propensity for mortality is pronounced. In the year 2022, with an average of 17 per 1000 live births, approximately 2.3 million neonates died worldwide, equating to about 6300 neonatal deaths daily [30]. Within the Indian context, current neonatal mortality rate is 22 deaths per 1000 live births, higher than the global mortality rate of 17. An estimated 26 million births occur annually, with children aged 0–6 years comprising 13% of the nation’s total population, according to the 2011 Census. India’s higher neonatal mortality rate, as reported by UNICEF, can be attributed to premature births, infections, and shortcomings in healthcare services, in addition to birth asphyxia, trauma, and congenital anomalies that contribute to nearly 40% of all deaths in children under the age of five [31]. While several governmental initiatives are working towards early identification of syndromic association in the prenatal and perinatal period, introducing essential pharmacogenomics in newborn screening promises the potential to improve neonatal health outcomes in the country. The lack of information for PGx evidence for several drugs underlines the need for continued research to strengthen the evidence base for neonatal drugs and careful consideration of adverse effects in neonatal drug therapy.

The AMP PGx Working Group’s classification of PGx alleles into Tier 1 (mandatory) and Tier 2 (optional) categories is a pivotal step in standardizing PGx testing across clinical laboratories. Overall, nine genes (*CYP2C19*, *CYP2C9*, *CYP2D6*, *TPMT*, *NUTD15*, *CYP3A4*, *CYP3A5*, *VKORC1* and *CYP4F2*) are suggested to be offered for every individual which plays a crucial role in the metabolism of 56 drugs. In India, with our genetic heterogeneity, this approach can be adapted. Variants in genes like *CYP2C19* and *CYP2D6* are prevalent in the Indian population and affect the metabolism of several drugs, which underscores the need for tailored PGx testing and the development of ethnic-specific panels, which could improve the efficacy of treatment regimens for chronic diseases. Adopting AMP’s evidence-based standards of testing a minimum of nine genes at the first level followed by an elaborate panel of 24 genes for essential drugs in India can enhance the quality and safety of medical interventions, offering personalized care suited to the genetic makeup of Indian patients.

From the WHO list of essential medicines, Ibuprofen and Omeprazole (Level 1 and 2 evidence, respectively) are sold in India over the counter. These drugs depend on the metabolism of *CYP2C9* and *CYP2C19* genes.

The pharmacogenomics landscape of the Indian population from the IndiGenomes data was published in 2022 [32]. It enlists several novel and deleterious variants associated with pharmacogenomics and estimated that every Indian harbors about eight variants that are medically actionable. Subsequently, the same group published the *CYP2D6* variant spectrum and pharmacogenomics of non-insulin antidiabetic drugs [33,34].

IndiGenomes project is limited by data availability, underrepresentation of diverse populations, and the complexity of integrating this data into existing frameworks.

We anticipate the release of extensive genetic information from projects like the 10,000 Genomes Project [35], which will expand our current understanding of genetic variation. This influx of data is expected to include revised frequencies that will enhance our ability to conduct more detailed studies of pharmacogenomic attributes. The novelty of this research lies in a comprehensive cross-sectional study that amalgamates three objectives: indigenous data facilitated by the WHO, drugs listed in the NICU medication list, and the AMP allele set. By comparing these with the available status of pharmacogenomic information, this scoping review aims to reinforce and redefine our understanding of pharmacogenomic interactions in diverse populations.

We have cataloged the list of genes responsible for the metabolism of essential drugs, drugs used in neonatal care, and some of the over-the-counter drugs available in India. We acknowledge the limitations of the study wherein we have cataloged the pharmacogenomics of essential drugs and collated information from Indian and international databases to propose the ideal pharmacogenomic assay for the Indian population; however, the actual markers required in clinical set up could be elaborate as well as may require further exploration. Clinical validation of the proposed assay along with healthcare outcomes is recommended to further iterate its utility. There are several drugs that have pharmacogenomic information characterized in other populations but not in India. With the PGx status of essential medications cataloged and with the available variant landscape of the Indian population from recent studies, we hope the clinical research involving pharmacogenomics will focus on comprehensive genotyping and functional/phenotypic correlation to develop an effective population-specific pharmacogenomics panel and practicing guidelines for India.

Based on these future developments, healthcare providers should embrace a minimum allele testing approach that reflects both global pharmacogenomic standards and localized genetic nuances. This proposed panel of 24 genes should be routinely tested to ensure that personalized medicine can be effectively implemented in the Indian clinical setting, with the potential to expand and adapt as new data becomes available. The alleles enlisted should be actively investigated in the clinical settings, included in translational clinical research, and should be made available for all individuals who undergo exome/genome analyses for other primary health ailments. Other variants identified in the 24 genes should also be reported and submitted in the public domain so that future functional assays, pharmacokinetics—pharmacodynamics studies and genotype–phenotype correlation investigations are informed. This forward-thinking strategy will augment the quality of patient care and serve as a model for pharmacogenomic applications in diverse populations worldwide.

## 6. Conclusions

We have cataloged the actionable pharmacogenomics of essential medicines and drugs used in neonatal intensive management along with their available Indian frequencies. Based on the current available clinically actionable guidelines, we propose pre-emptive testing of a 24 gene panel to assist clinicians in delivering personalized medicine. Based on the variant/allele spectrum in these 24 genes, future translational studies can be informed, paving the way for developing personalized medicine in the Indian healthcare setup.

## Figures and Tables

**Table 1:** WHO list of essential medicines along with PharmGKB associated genes with CPIC 1 and 2 level evidence polymorphisms and IndiGen frequency.

ADULTS			
CONDITION	DRUG	GENE	STAR ALLEL/rsID
HIV	Atazanavir	*UGT1A1*	*1, *6, *80
	Efavirenz	*CYP2B6*	*4, *6, *7, *8, *9, *12, *13*18, *19, *20, *26, *34, *36, *37, *38, *39, *40, *41, *42, *43,
	Abacavir	*HLA-B*	*57:01
Hypertension	Metoprolol	*CYP2D6*	*4, *10,*31,*6,,*9,*29,*3, 5, *161, *156, *144, *143, *129, *124, *120, *114, *101, *100, *99, *96, *92, *81, *69, *62, *60, *56, *51, *47, *44, *42, *38, *36, *21, *20, *19, *18, *15, *12
Malignancy	Capecitabine Fluorouracil	*DPYD*	rs75017182, rs3918290
	Irinotecan	*UGT1A1*	*6, *80
	Mercaptopurine	*NUDT15*	*1, *2, *3,
		*TPMT*	*1, *3A, *3B, *3C, *41
	Tamoxifen	*CYP2D6*	*1, *2, *4, *6, *10, *17, *29, *41, *3, *5, *7, *8, *9, *11, *21, *36, *12, *13, *14, *15, *19, *20, *27, *31, *32, *40, *42, *44, *47, *49, *51, *54, *55, *56
Cardiovascular disease	Clopidogrel	*CYP2C19*	*2, *3, *4, *5, *6, *8, *10, *19, *25, *26, *7, *9, *16, *22, *24, *35, *36, *37
	Lovastatin Atorvastatin Simvastatin Pravastatin	*SLCO1B1*	*1, *5, *15, *31, *46, *47
	Fluvastatin	*SLCO1B1*	*1, *5, *15, *31, *46, *47
		*CYP2C9*	*1, *2, *3, *8, *11, *14, *26, *35, *44, *45, *61
	Warfarin	*CYP2C9*	*1, *2, *3
		*VKORC1*	rs9923231
Hepatitis	Ribavirin	*IFNL4*	rs12979860
Psychotropic medications	Amitriptyline	*CYP2C19*	*1, *2, *3, *17, *4, *5, *6, *7, *8, *22, *24, *35
		*CYP2D6*	*1,*2,*4,*9,*10,*17, *161, *156, *144, *143, *132, *129, *124, *119, *114, *109, *101, *100, *99, *91, *81, *69, *62, *60, *59, *56, *55, *54, *52, *51, *50, *49, *47, *45, *44, *42, *41, *40,*36, *35, *31, *29, *21, *19, *18, *15, *14, *13, *12, *11, *8, *7, *6, *5, *3, *27, *32
	Clomipramine	*CYP2C19*	*1, *2, *3, *17, *4, *5, *6, *7, *8, *22, *24, *35
		*CYP2D6*	*1,*2,*4,*9,*10,*17, *161, *156, *144, *143, *132, *129, *124, *119, *114, *109, *101, *100, *99, *91, *81, *69, *62, *60, *59, *56, *55, *54, *52, *51, *50, *49, *47, *45, *44, *42, *41, *40,*36, *35, *31, *29, *21, *19, *18, *15, *14, *13, *12, *11, *8, *7, *6, *5, *3, *27, *32
	Sertraline	*CYP2B6*	*6, *9, *7, *8, *12, *13, *18, *19, *20, *43, *42, *41, *40, *39, *38, *37, *36, *34, *28, *26
		*CYP2C19*	*2, *3, *35, *26, *25, *24, *22, *19, *16, *10, *9, *8, *7, *6, *5, *4, *17
	Aripiprazole	*CYP2D6*	*4, *6, *114, *42, *40, *38, *36, *31, *21, *20, *19, *18, *13, *12, *11, *8, *7, *5, *3, *15
	Haloperidol		*114, *42, *40, *38, *36, *35, *31, *21, *20, *19, *18, *15, *13, *12, *11, *8, *7, *6, *4, *5, *3, *2, *1
	Zuclopenthixol decanoate		*114, *42, *41, *40, *38, *36, *35, *31, *29, *21, *20, *19, *18, *17, *15, *14, *13, *12, *11, *10, *9, *8, *7, *6, *5, *4, *3, *2, *1
	Fluvoxamine		*161, *156, *144, *143, *129, *124, *114, *101, *100, *99, *96, *81, *69, *62, *60, *56, *51, *47, *44, *42, *40, *38, *36, *31, *21, *20, *19, *18, *15, *13, *12, *11, *8, *7, *6, *5, *4, *3
	Paroxetine		*161, *156, *144, *143, *132, *129, *124, *119, *114, *109, *101, *100, *99, *96, *91, *81, *69, *62, *60, *59, *55, *54, *52, *51, *50, *49, *47, *45, *44, *42, *41, *40, *38, *36, *35, *32, *31, *29, *27, *21, *20, *19, *18, *17, *15, *14, *13, *12, *11, *10, *9, *8, *7, *6, *5, *4, *3, *2, *1
	Risperidone		*114, *42, *40, *38, *36, *35, *31, *21, *20, *19, *18, *15, *13, *12, *11, *8, *7, *6, *5, *4, *3, *2, *1
	Bupropion	*CYP2D6*	*2, *3, *4, *5, *6, *9, *10, *17, *29, *40, *41 (FDA recommendation for poor metabolizers)
	Carbamazepine	*HLA-A*	*31:01
		*HLA-B*	*15:02
	Citalopram, Escitalopram	*CYP2C19*	*2, *3, *4, *17, *35, *26, *25, *24, *22, *19, *16, *10, *9, *8, *7, *6, *5, *18
	Phenytoin	*CYP2C9*	*2, *3, *8, *11, *14, *26, *35, *44, *45, *61
		*HLA-B*	*15:02
	Lamotrigine	*HLA-B*	*15:02
	Quetiapine	*CYP3A4*	*13, *22
Immunosuppressive medications	Azathioprine	*TPMT*	*1, *3A, *3B, *3C, *8, *41,
		*NUDT15*	*1, *2, *3,
Analgesics	Codeine, Tramadol	*CYP2D6*	*1,*2, *3,*4,*5,*6,*7,*8,*9,*10,*161, *156, *144, *143, *132, *129, *124, *114, *109, *101, *100, *99, *96, *91, *56, *81, *69, *62, *59, *55, *54, *52, *51, *49, *47, *45,*44, *42, *41, *40, *38, *36, *35, *32, *31, *29, *27, *21, *20, *19, *18, *17, *15, *14, *13, *12, *11
Tuberculosis	Isoniazid	*NAT2*	*5, *6, *7, *14, *16
Drug hypersensitivity	Allopurinol	*HLA-B*	*58:01
		*ABCG2*	rs2231142
	Dapsone	*G6PD*	FDA recommendation
Gastroesophageal reflux disease (GERD)	Omeprazole ^otc^	*CYP2C19*	*1, *2, *3, *17, *9, *38, *35, *28, *26, *25, *24, *22, *19, *18, *16, *15, *13, *11, *10, *8, *7, *6, *5, *4
Antiemetics	Tropisetron, Ondansetron	*CYP2D6*	*1, *2, *13, *27, *35, *45
Immunodeficiency	Tacrolimus	*CYP3A5*	*1, *3
Fungal or yeast infections	Voriconazole	*CYP2C19*	*2, *3, *4, *17, *35, *24, *22, *8, *7, *6, *5
**PEDIATRIC**			
Psychotropic medications	Phenytoin	*CYP2C9*	CYP2C9 *1, *2, *3, *8, *9, *11, *14, *26, *35, *44, *45
Immunosuppressive medications	Azathioprine	*NUDT15*	*1, *2, *5, *3
		*TPMT*	*1, *3A, *3B, *3C, *22, *34, *41
Malignancy	Fluorouracil	*DPYD*	rs2297595, rs56038477, rs1801158, rs1801160, rs75017182
	Mercaptopurine	*NUDT15*	*1, *2, *3 *5
		*TPMT*	*1, *3A, *3B, *3C, *8, *16
	Methotrexate	*MTHFR*	rs1801133
	Irinotecan	*UGT1A1*	*1, *6, *80+*28, *80+*37 rs4124874
Inflammatory diseases	Ibuprofen ^otc^	*CYP2C9*	*1, *2, *3 *8, *9, *11, *14, *26, *35, *37, *39, *42, *43, *44, *45, *46, *52, *55, *61
Toxic liver disease	Isoniazid	*NAT2*	*5, *6, *7, *14, *16
Gastroesophageal reflux disease (GERD)	Omeprazole ^otc^	*CYP2C19*	*1, *2, *3, *9, *10, *17, *4, *5, *6, *7, *8, *10, *11, *13, *15, *16, *18, *19, *22, *25, *26, *28, *35, *38 *24
Antiemetics	Ondansetron	*CYP2D6*	*1, *2, *4, *10, *35 *14, *17, *27, *29, *33, *34, *39, *45
Hepatitis virus	Ribavirin	*IFNL3, IFNL4*	rs12979860, rs8099917
General anaesthetics	Sevoflurane	*RYR1*	rs193922809
	Halothane, Isoflurane	*RYR1*	rs118192161, rs118192162, rs193922772, rs112563513, rs193922816, rs28933397, rs118192122, rs193922747, rs118192176, rs118192177, rs118192175, rs121918592, rs121918593, rs118192172 rs121918594
		*CACNA1S*	rs1800559, rs772226819
Immunosuppressive agents	Tacrolimus	*CYP3A4,*	*1, *22
		*CYP3A5*	*1, *3, *6, *7
Fungal infections	Voriconazole	*CYP2C19*	*1, *2, *3, *17, *4, *5, *6, *7, *8, *9, *10, *16, *17, *19, *22, *24, *25, *26, *35
Cardiovascular medications	Warfarin	*CYP2C9*	*1, *2, *3, *8, *11
		*CYP4F2*	rs2108622
		*VKORC1*	rs9923231, rs7294, rs9934438, rs2359612, rs8050894, rs9923231
Drug hypersensitivity	Allopurinol	*HLA B*	*58:01

* = STAR ALLELE. otc—Over-the-counter drugs available in India.

**Table 2:** Drugs used in NICU as per London Health Sciences Centre (LHSC), IOWA Health Care, Medication Use in Neonatal Intensive Care Unit (2005–2018) in the United States, and the Association of Neonatal Nurses (NANN): list of drug-gene pair along with IndiGen frequency.

DRUG	SPECIALITY PURPOSE	ADVERSE REACTIONS	PHARMGKB GENE	STAR ALLELE	rsID	INDIGEN
Lansoprazole	Gastroesophageal-reflux-disease (GERD)	GI: abdominal pain, cramps, bloating, constipation, diarrhoea, vomiting, mildly elevated AST/ALT CV: hypertension, hypotension, Dermatologic: urticaria, pruritus, Hematologic: thrombocytopenia, leucopenia, leukocytosis, anemia, Endocrine: hyperglycemia, hyperlipidemia	*CYP2C19*	*1	rs3758581	0.8889
				*2	rs12769205	0.3689
					rs4244285	0.3678
					rs5897349	0.0025
				*3	rs4986893	0.0064
				*8	rs41291556	NA
					rs3758581	0.8889
				*9	rs17884712	NA
					rs3758581	0.8889
				*17	rs12248560	0.1436
Pantoprazole	Gastroesophageal-reflux-disease (GERD)	abdominal pain, cramps, bloating, constipation, diarrhea, vomiting, hypertension, hypotension, urticaria, pruritus, thrombocytopenia, leucopenia, leukocytosis, anemia, thrombophlebitis	*CYP2C19*	*1	rs3758581	0.8889
				*2	rs12769205	0.3689
					rs4244285	0.3678
					rs58973490	0.0025
				*3	rs4986893	0.0064
				*8	rs41291556	NA
					rs3758581	0.8889
				*9	rs17884712	NA
					rs3758581	0.8889
				*17	rs12248560	0.1436
phenytoin	Anticonvulsant	Acute, following IV administration: hypotension, bradycardia, ventricular fibrillation, vasodilation; venous irritation; pain, thrombophlebitis, skin rash. Observe IV site carefully. Extravasation may cause tissue inflammation and necrosis. GI side effects: vomiting, constipation. Other: toxic hepatitis, gingival hyperplasia, hyperglycemia and osteoporosis	*CYP2C9*	*1	rs72558189	0.001
					rs200965026	0.0049
					rs199523631	0.0005
					rs1799853	0.0186
					rs17847037	0.0015
					rs7900194	0.0010
					rs2256871	0.0103
					rs28371685	0.0152
					rs1057910	0.0182
				*2	rs1799853	0.0307
				*3	rs1057910	0.1093
				*5	rs28371686	NA
				*6	rs9332131	NA
				*8	rs7900194	0.0005
				*11	rs28371685	0.0029
				*13	rs72558187	NA
				*14	rs72558189	0.018
				*16	rs72558192	NA
				*29	rs182132442	NA
				*31	rs57505750	NA
				*33	rs200183364	NA
				*37	rs564813580	NA
				*39	rs762239445	NA
				*42	rs12414460	NA
				*43	rs767576260	NA
				*45	rs199523631	0.0015
				*50	NA	NA
				*52	rs988617574	NA
				*55	rs1250577724	NA
Gentamicin	Antibiotic	Ototoxicity, Nephrotoxicity	*MT-RNR1*		rs267606618	NA
					rs267606619	NA
Tobramycin	Antibiotic	Ototoxicity, Nephrotoxicity	*MT-RNR1*		rs267606617	NA
					rs267606619	NA
Amikacin	Antibiotic	Ototoxicity, nephrotoxicity	*MT-RNR1*		rs267606617	NA
Omeprazole	Gastroesophageal-reflux-disease (GERD)	gastrointestinal disturbances such as diarrhoea, abdominal discomfort, and occasionally, an increased risk of infections due to altered gastric pH levels	*CYP2C19*	*1	rs3758581	0.8889
				*2	rs12769205	0.3689
				*2	rs4244285	0.3678
				*2	rs58973490	0.0025
				*3	rs4986893	0.0064
				*9	rs17884712	NA
				*9	rs3758581	0.8889
				*10	rs6413438	NA
				*10	rs3758581	0.8889
				*17	rs12248560	0.1436
				*24	rs3758581	0.8889
				*24	rs118203757	NA
Succinylcholine	Muscle Relaxant	Hyperkalemia, Malignant Hyperthermia, Bradycardia, Increased Intracranial Pressure, Myoglobinuria	*RYR1*		rs193922802	NA
			*RYR1*		rs193922816	NA
			*RYR1*		rs112563513	NA
			*RYR1*		rs121918596	NA
			*CACNA1S*		rs1800559	NA
			*RYR1*		rs121918592	NA
			*RYR1*		rs118192122	NA
			*RYR1*		rs193922772	NA
					rs118192163	NA
					rs118192177	NA
					rs118192176	NA
					rs118192124	NA
					rs121918595	NA
					rs28933397	NA
					rs118192178	NA
					rs121918593	NA
					rs1801086	NA
					rs193922807	NA
					rs118192168	NA
					rs118192172	NA
					rs193922876	NA
					rs193922764	NA
					rs193922818	NA
Epinephrine	Sympathomimetic drug (relaxing muscles)/catecholamines	Breathing problems, irregular heartbeats, pale skin, sweating, nausea, vomiting, dizziness, tremors, headache, restlessness, fear, nervousness, anxiety, excitation	*G6PD* Deficiency	NA	NA	NA
Ibuprofen	Anti inflammatory	Headache, dizziness, drowsiness, fatigue, restless sleep, thirst, sweating, numbness in hands and feet, impaired hearing, blurred vison, eye irritation, fluid retention, ankle swelling, mild allergic reaction, abdominal pain, nausea, vomiting, heat burn, diarrhoea, constipation, frequent urination, bladder irritation, increase risk of heart attack or stroke, bleeding in stomach and bowels, kidney and liver damage, confusion, disorientation, tinnitus, anxiety, paranoia, anaemia, black stools, seizures, coma	*CYP2C9*	*1	rs72558189	0.001
					rs200965026	0.0049
					rs199523631	0.0005
					rs1799853	0.0186
					rs17847037	0.0015
					rs7900194	0.0010
					rs2256871	0.0103
					rs28371685	0.0152
					rs1057910	0.0182
				*2	rs1799853	0.0307
				*3	rs1057910	0.1093
				*8	rs7900194	0.0005
				*9	rs2256871	0.0817
				*11	rs28371685	0.0029
				*14	rs72558189	0.018
				*26	rs200965026	0.0049
				*35	rs72558189	0.001
					rs1799853	0.0186
				*37	rs564813580	NA
				*39	rs762239445	NA
				*42	rs12414460	NA
				*43	rs767576260	NA
				*44	rs200965026	0.0049
				*45	rs199523631	0.0015
				*46	rs754487195	NA
				*52	rs988617574	NA
				*55	rs1250577724	NA
				*61	rs202201137	NA
					rs1799853	0.0186
Metoclopramide	Dopamine receptor antagonist	Tardive dyskinesia, diarrhoea, drowsiness, fatigue, muscle pain, restlessness, parkinsonism, somnolence, nausea, vomiting, asthenia, lassitude, depression, hypotension	*CYP2D6*	Poor metabolizers (*2, *3, *4, *5, *6, *9, *10, *17, *29, *40, *41)	rs1058164	0.568
					rs16947	0.374
					rs1135840	0.5646
					rs28371725	0.1324
					rs5030656	0.0020
					rs5030655	NA
					rs28371704	0.0884
					rs3892097	0.1094
					rs1058172	0.0781
					rs1135832	NA
					rs1135833	NA
					rs35742686	NA
					rs61736512	NA
					rs59421388	NA
					rs1135835	NA
					rs1135836	NA
					rs74478221	NA
					rs766507177 rs1065852	NA
						0.1929
					rs28371703	0.0894
					rs28371735	NA
					rs1135824	0.0010
					rs72549356	NA
					rs28371706	NA
					rs28371736	NA
					rs747998333	NA
					rs75467367	NA

* = STAR ALLELE. NA—IndiGen data not available.

**Table 3:** AMP’s Minimum Set of Alleles along with different population frequencies from the IndiGen database.

TIER 1* MINIMUM SET						
GENE	ALLELE	rsID	IndiGen	African	Asian (East & South)	Europe
*CYP2C19*	*2	rs12769205	0.3689	0.1967	0.3125 & 0.3579	0.1451
		rs4244285	0.3678	0.1702	0.3125 & 0.3579	0.1451
		rs58973490	0.0025	0.0008	0 & 0	0.0040
	*3	rs4986893	0.0064	0.0023	0.0556 & 0.0123	0.000
	*17	rs12248560	0.1436	0.2352	0.0149 & 0.136	0.2237
*CYP2C9*	*2	rs1799853	0.0307	0.0083	0.001 & 0.0348	0.1243
	*3	rs1057910	0.1093	0.0023	0.0337 & 0.1094	0.0726
	*5	rs28371686	NA	NA	NA	NA
	*6	rs9332131	NA	NA	NA	NA
	*8	rs7900194	0.0005	0.053	0.0 & 0.001	0.002
	*11	rs28371685	0.0029	0.0242	0.0 & 0.001	0.002
*CYP2D6*	*2	rs1058164	0.568	NA	NA	NA
	*4	rs3892097	0.1094	0.0605	0.002 & 0.1094	0.1859
	*5	NA	NA	NA	NA	NA
	*6	rs5030655	NA	0.0008	0.0000	0.0199
		rs1135840	0.5646	NA	NA	NA
	*9	rs5030656	0.002	0.0008	0.0000	0.0258
	*10	rs1065852	0.1929	0.1127	0.5714	0.2018
		rs1058164	0.568	NA	NA	NA
	*17	rs1058164	0.568	NA	NA	NA
		rs16947	0.374	NA	NA	NA
		rs1135840	0.5646	NA	NA	NA
	*29	rs61736512	NA	0.1097	0.0000	0.0000
		rs1058164	0.568	NA	NA	NA
		rs16947	0.374	NA	NA	NA
		rs59421388	NA	0.1074	0.0000	0.0000
		rs1135840	0.5646	NA	NA	NA
	*41	rs28371725	0.1324	0.0182	0.0377	0.9066
*TPMT*	*2	rs1800462	NA	NA	NA	NA
	*3A	rs1800460	0.0039	0.003	0.0041	0.0278
		rs1142345	0.0226	0.0666	0.0218 & 0.0174	0.0288
	*3B	rs1800460	0.0039	0.003	0.0041	0.0278
	*3C	rs1142345	0.0226	0.0666	0.0218 & 0.0174	0.0288
*NUDT15*	*3	rs116855232	0.0837	0.0008	0.0952 & 0.0695	0.002
*CYP3A4*	*22	rs35599367	0.0083	0.0008	0.0061	0.0497
*CYP3A5*	*3	rs776746	0.7059	NA	NA	NA
	CYP3A5*6	rs10264272	NA	NA	NA	NA
	CYP3A5*7	rs41303343	NA	NA	NA	NA
TIER 2* OPTIONAL						
*CYP2C19*	*4	rs12248560	0.1436	0.2352	0.0149 & 0.136	0.2237
	*5	rs3758581	0.8889	NA	NA	NA
	*6	rs72552267	NA	NA	NA	NA
		rs3758581	0.8889	NA	NA	NA
	*7	rs3758581	0.8889	NA	NA	NA
		rs72558186	NA	NA	NA	NA
	*8	rs41291556	NA	NA	NA	NA
		rs3758581	0.8889	NA	NA	NA
	*9	rs17884712	NA	NA	NA	NA
		rs3758581	0.8889	NA	NA	NA
	*10	rs6413438	NA	NA	NA	NA
		rs3758581	0.8889	NA	NA	NA
	*35	rs17882687	NA	NA	NA	NA
		rs12769205	0.3689	0.1967	0.3125 & 0.3579	0.1451
		rs3758581	0.8889	NA	NA	NA
*CYP2C9*	*12	rs9332239	NA	NA	NA	NA
	*13	rs72558187	NA	NA	NA	NA
	*14	rs72558190	NA	NA	NA	NA
*CYP4F2*	*3	rs2108622	0.4012	0.0825	0.2143 & 0.4131	0.2903
*VKORC1*		rs72547529	NA	NA	NA	NA
		rs61742245	NA	NA	NA	NA
*CYP2C* cluster		rs12777823	0.3741	0.2511	0.3145 & 0.362	0.1511
*CYP2D6*	*7	rs5030867	0.0073	NA	0.0 & 0.0092	NA
	*8	rs1058164	0.5680	NA	NA	NA
		rs5030865	NA	NA	NA	NA
		rs16947	0.374	NA	NA	NA
		rs1135840	0.5646	NA	NA	NA
	*12	rs5030862	NA	NA	NA	NA
		rs1058164	0.5680	NA	NA	NA
		rs28371710	NA	NA	NA	NA
		rs16947	0.374	NA	NA	NA
		rs1135840	0.5646	NA	NA	NA
	*14	rs1058164	0.5680	NA	NA	NA
		rs5030865	NA	NA	NA	NA
		rs16947	0.3740	NA	NA	NA
		rs1135840	0.5646	NA	NA	NA
	*15	rs28371696	0.0074	0.0234	0.0 & 0.0112	0.002
		rs774671100	NA	NA	NA	NA
	*21	rs1058164	0.568	NA	NA	NA
		rs16947	0.374	NA	NA	NA
		rs1135840	0.5646	NA	NA	NA
	*31	rs16947	0.374	NA	NA	NA
		rs1135840	0.5646	NA	NA	NA
		rs1058164	0.5680	NA	NA	NA
	*40	rs72549356	NA	NA	NA	NA
	*42	rs1058164	0.5680	NA	NA	NA
		rs16947	0.374	NA	NA	NA
		rs72549346	NA	NA	NA	NA
		rs1135840	0.5646	NA	NA	NA
	*49	rs1065852	0.1929	0.1127	0.5714 & 0.1646	0.2018
		rs1135822	NA	NA	NA	NA
		rs1058164	0.5680	NA	NA	NA
		rs1135840	0.5646	NA	NA	NA
	*56	rs1065852	0.1929	0.1127	0.5714 & 0.1646	0.2018
		rs1058164	0.568	NA	NA	NA
		rs16947	0.374	NA	NA	NA
		rs72549347	NA	NA	NA	NA
		rs1135840	0.5646	NA	NA	NA
	*59	rs1058164	0.5680	NA	NA	NA
		rs16947	0.374	NA	NA	NA
		rs79292917	NA	NA	NA	NA
		rs1135840	0.5646	NA	NA	NA
*TPMT*	*11	rs72552738	NA	NA	NA	NA
	*29	rs267607275	NA	NA	NA	NA
	*42	rs759836180	NA	NA	NA	NA
*NUDT15*	*2	rs746071566	NA	NA	NA	NA
		rs116855232	0.0837	0.0008	0.0952 & 0.0695	0.002
	*4	rs147390019	NA	NA	NA	NA
	*6	rs746071566	NA	NA	NA	NA
	*9	rs746071566	NA	NA	NA	NA
	*14	rs777311140	NA	NA	NA	NA
*CYP3A4*	*20	rs67666821	NA	NA	NA	NA

* = STAR ALLELE. NA—data not available in IndiGenomes.

**Table 4:** List of 24 genes and associated drugs that we propose for pre-emptive testing.

Sl. No	Genes	Drugs	Sl. No	Genes	Drugs
1	*CYP2D6*	Metoprolol, Tamoxifen Amitriptyline, Clomipramine Ondansetron, Tropisetron Codeine, Tramadol Bupropion, Aripiprazole Haloperidol, Fluvoxamine Zuclopenthixol decanoate Paroxetine, Risperidone	13	*NUDT15*	Mercaptopurine Azathioprine
2	*VKORC1*	Warfarin	14	*DPYD*	Fluorouracil Capecitabine
3	*TPMT*	Mercaptopurine Azathioprine	15	*CYP2C9*	Fluvastatin, Warfarin Phenytoin, Ibuprofen
4	*NAT2*	Isoniazid	16	*CYP2B6*	Sertraline, Efavirenz
5	*HLA-A*	Carbamazepine	17	*CYP2C19*	Clopidogrel, Amitriptyline Clomipramine, Sertraline Citalopram, Escitalopram Omeprazole, Voriconazole
6	*HLA-B*	Abacavir, Phenytoin Lamotrigine, Allopurinol	18	*MTHFR*	Methotrexate
7	*G6PD*	Dapsone	19	*IFNL3*	Ribavirin
8	*CYP3A4*	Tacrolimus, Quetiapine	20	*IFNL4*	Ribavirin
9	*CYP3A5*	Tacrolimus	21	*CACNA1S*	Halothane, Isoflurane
10	*ABCG2*	Allopurinol	22	*RYR1*	Sevoflurane, Halothane Isoflurane
11	*UGT1A1*	Atazanavir, Irinotecan	23	*CYP4F2*	Warfarin
12	*SLCO1B1*	Lovastatin, Atorvastatin Simvastatin, Pravastatin Fluvastatin	24	*MT-RNR1*	Gentamicin, Tobramycin Amikacin

Blue—common for adults and pediatrics, Green—unique to pediatrics, Black highlight—unique to adults.

## Data Availability

The datasets generated and/or analyzed during the current study are available in the PharmGKB (https://www.pharmgkb.org/, accessed on 10 March 2024) and IndiGenomes (https://clingen.igib.res.in/indigen/index.php, accessed on 10 March 2024) websites.

## Supplementary Materials

The following supporting information can be downloaded at: https://www.mdpi.com/article/10.3390/jox15040101/s1. Supplementary Table S1: Essential Drugs from WHO: PharmGKB data and the results of adult and pediatric actionable drug–gene pair along with global frequencies; Supplementary Table S2: NICU drug list and associated adverse drug reactions along with Level 1 and Level 2 pharmacogenomic evidence; Supplementary interactive Graph S1: HTML file for drugs with CPIC Level 1 and 2 evidence for adults with associated genes and medical conditions; Supplementary interactive Graph S2: HTML file for drugs with CPIC Level 1 and 2 evidence for adults with associated genes and medical conditions; Supplementary interactive Graph S3: HTML file for identified adverse effects of NICU drugs.

## Abbreviations

PGx	Pharmacogenomics
PharmGKB	Pharmacogenomics Knowledge Base
PGRN	Pharmacogenomics Research Network
UPGx	Ubiquitous Pharmacogenomics Consortium
ISPG	International Society of Pharmacogenomics
FDA	Food and Drug Administration
CPIC	Clinical Pharmacogenetics Implementation Consortium
DPWG	Dutch Pharmacogenetics Working Group
MAF	Minor allele frequency
WHO	World Health Organization
NICU	Neonatal Intensive Care Unit
LHSC	London Health Sciences Centre
AMP	Association for Molecular Pathology
SNP	Single Nucleotide Polymorphism
rsID	Reference SNP identifiers
PPI	Proton Pump Inhibitors
ADRs	Adverse drug reactions
AST	Aspartate Transaminase
ALT	Alanine Transaminase
HIV	Human immunodeficiency viruses
UNICEF	United Nations International Children’s Emergency Fund
